# ZODET: Software for the Identification, Analysis and Visualisation of Outlier Genes in Microarray Expression Data

**DOI:** 10.1371/journal.pone.0081123

**Published:** 2014-01-08

**Authors:** Daniel L. Roden, Gavin W. Sewell, Anna Lobley, Adam P. Levine, Andrew M. Smith, Anthony W. Segal

**Affiliations:** Division of Medicine, University College London, London, United Kingdom; The Scripps Research Institute, United States of America

## Abstract

**Summary:**

Complex human diseases can show significant heterogeneity between patients with the same phenotypic disorder. An outlier detection strategy was developed to identify variants at the level of gene transcription that are of potential biological and phenotypic importance. Here we describe a graphical software package (z-score outlier detection (ZODET)) that enables identification and visualisation of gross abnormalities in gene expression (outliers) in individuals, using whole genome microarray data. Mean and standard deviation of expression in a healthy control cohort is used to detect both over and under-expressed probes in individual test subjects. We compared the potential of ZODET to detect outlier genes in gene expression datasets with a previously described statistical method, gene tissue index (GTI), using a simulated expression dataset and a publicly available monocyte-derived macrophage microarray dataset. Taken together, these results support ZODET as a novel approach to identify outlier genes of potential pathogenic relevance in complex human diseases. The algorithm is implemented using R packages and Java.

**Availability:**

The software is freely available from http://www.ucl.ac.uk/medicine/molecular-medicine/publications/microarray-outlier-analysis.

## Introduction

Many human diseases, such as inflammatory bowel disease and type 1 diabetes, are complex, multifactorial syndromes with genetic and environmental determinants. Substantial heterogeneity with regards to causation and disease progression exists between individual patients with the same phenotypic disorder. It has been postulated that rare or low frequency variants, structural rearrangements such as deletions, insertions, translocations, and epigenetic variation could be important in the pathogenesis of these complex disorders and account for the observed heterogeneity [1]. All of these are incompletely assessed by current genome wide association studies (GWAS). Many of these genetic changes would be expected to be associated with alterations in gene expression, possibly of large biological effect, ultimately giving rise to phenotypic abnormalities. A recent paper combined population-scale human genomic sequence data with transcriptomic data and identified an enrichment of rare variants associated with outlier gene expression [2]. They concluded that across multiple tissues and developmental stages, an individual would be expected to have hundreds of rare variants with large effects on gene expression. The examination of significantly over-expressed genes in individual patients (or subgroups of patients) has successfully been employed in the field of cancer genomics [3]. There are a number of methods for outlier detection currently in the literature, such as the gene tissue index (GTI), cancer outlier profile analysis (COPA) and outlier robust test (ORT), each of which use different algorithms in order to identify probes that are abnormally expressed in subgroups of patients [4]–[6]. Here we describe a software package based on z-score outlier detection (ZODET) that enables identification of potentially biologically relevant abnormalities in gene expression (outliers) in individuals with complex disorders compared with a comparison population, using whole genome microarray data. By concentrating on individual outliers, we provide a valuable addition to commonly used microarray analysis tools, such as SAM [7].

## Materials and Methods

### Software Implementation

**Figure 1:**
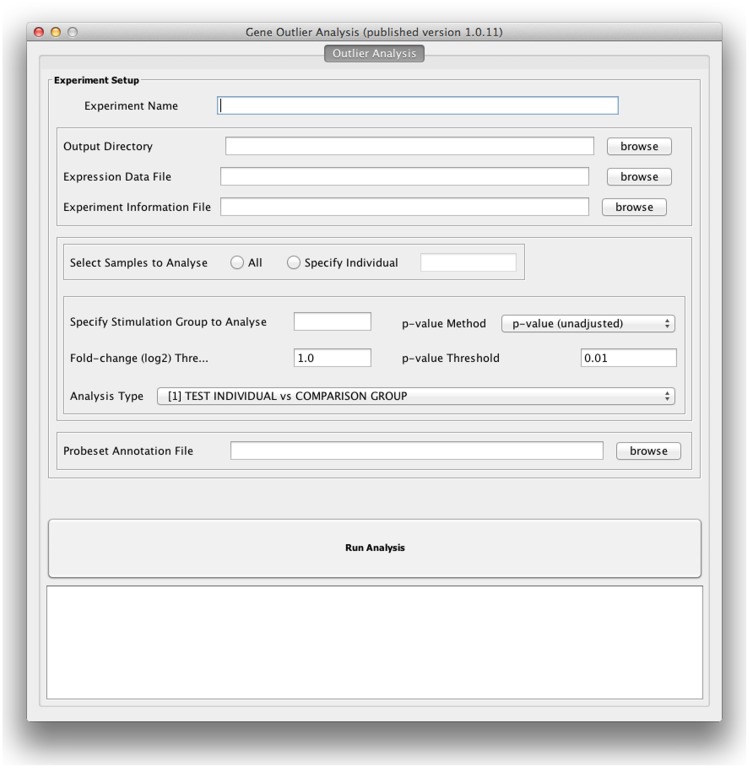
The Graphical User Interface (GUI) allows the user to set the analysis parameters, the required fold change, statistical test (p-value, q-value or Bonferroni corrected p-value) and statistical threshold. The analysis can be conducted using the whole experimental group or a specific individual can be chosen.

Implementation of this software has used two programming technologies: The R statistical programming environment (http://www.R-project.org), utilising the *Biobase* package from the Bioconductor platform; and the Java programming language [8]. The analysis can be run via a configurable Graphical User Interface (GUI) or on the command line (Figure 1). Installation and configuration instructions are provided in the technical documentation supplied with the software (http://www.ucl.ac.uk/medicine/molecular-medicine/publications/microarray-outlier-analysis). The software is freely available and can be run on either Windows or Mac OSX operating systems.

**Figure 2:**
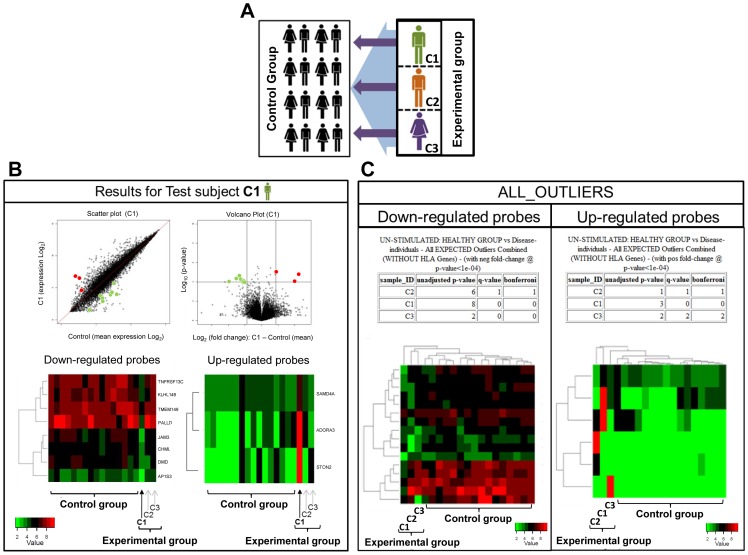
Overview of experimental design and output from the ZODET analysis. **A.** Microarray experimental design will contain two groups, a control group which will provide the data for the normal distribution of each probe and an experimental group (C1–3). The software runs each test group member independently against the control group and identify probes that are expressed at levels significantly outside the normal distribution. **B.** For each experimental subject the software generates a scatter plot of all of the expressed probes from subject C1 against the mean value from the control group. Probes that are classified as up- or down-regulated are highlighted in red and green, respectively. A Volcano plot is generated to visualise p-value and fold change information for all probes, with over and under expressed outliers highlighted in red and green, respectively. The vertical and horizontal lines represent the fold-change and p-value thresholds used, respectively. A combined dendrogram and heatmap shows the expression level of probes identified as down-regulated in C1, compared to the control group and two additional experimental subjects (C2 and C3). **C.** The software also generates outlier analysis results for all experimental subjects (C1–3). The total up-regulated and down-regulated probes are tabulated for all test subjects along with the results for the three available statistical tests. Combined dendrogram and heatmaps are generated for all of the up-regulated and down-regulated probes identified and hierarchical clustering on both probes and subjects performed. Over and under expressed probes are highlighted in red and green, respectively.

The purpose of this software is the identification and visual analysis of outlier probes (or genes) from microarray gene expression data. To identify the potential outliers a control group of samples and the experimental samples are defined prior to analysis (Figure 2A). Currently, the software supports five alternative methods for defining which sub-set of individual samples the potential outliers may occur in and the group of samples (control group) which each is compared to. These five methods of comparison are:

1. Test Individual vs Control Group
2. Test Individual vs All Samples
3. Test Individual vs Test Group
4. Control Individual vs Control Group
5. Control Individual vs All Samples

For each of the samples selected for outlier analysis, an iterative procedure is carried out on the expression data. Two adjustable thresholds (which both have to be met) are used to identify the probe outliers: (i) the significance level of the standardised deviation of the expression levels from the experimental sample, when compared to the average (mean) expression levels of the control group; and (ii) the (log_2_) fold-change between the expression level from the experimental sample and the average of the control group. This method assumes that the expression values are normally distributed. Therefore, caution should be taken to ensure the normality of the data, especially when using small sample sizes (e.g., less than 30).

To assess the significance of the standardised deviation of the experimental sample expression values from the control group average, a Z-score is calculated for each probe (or feature) on the microarray:
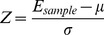
(1)Where: *E_sample_* is the expression level of the microarray gene probe for the experimental sample; and *μ* and *σ* are the mean and standard deviation of the expression levels of the gene probe for samples in the control group. From the Z-scores, p-values are then calculated with reference to a standard normal distribution where the p-value is the probability that the probe z-score of the sample being tested falls within the distribution of the control group samples. These p-values - or those corrected for multiple hypothesis testing, on a per gene basis, using either the Bonferroni or q-value method - can be used to assess the significance [9]. The default p-value threshold is 0.01, but this can be easily modified before each analysis run.

The fold-change between the expression level of the experimental sample, and the control group, is calculated for each probe on the array using the following method.
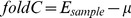
(2)


This provides an additional measure of the difference in the expression level of the experimental and the control groups and also determines whether the potential outlier-probe is under or over expressed, relative to the control group. In *equation 2*: *foldC* is the fold-change; *E_sample_* is the expression level of the test sample; and *μ* is the mean expression level of the control group. To correctly estimate the fold-change between sample classes all gene expression levels used as input to this software should be in normalised log_2_ format. The software is designed to not be microarray platform specific and can be used to analyse any log_2_ transformed microarray expression data (e.g., from Affymetrix, Illumina or other arrays). The default fold-change threshold is 1, which can be easily modified before each analysis run. User defined annotation files can also be used to associate the microarray probes with the relevant gene descriptions.

To aid the interpretation and visualisation of the results a number of clearly formatted graphs and tables are automatically generated by the software during the analysis, including:

For each individual (Figure 2B):

- Scatter plot of the correlation between the individual and mean control group expression values, with under and over expressed outlier probes colored green and red respectively (Figure 2B);
- Volcano plot (Figure 2B) highlighting the detected outlier probes: with fold-change and −log_10_(p-value) on the horizontal and vertical axes, respectively. Under and over expressed outlier-probes are colored green and red respectively;
- Heatmap of outlier probes for each individual (Figure 2B): the probes are clustered on the outlier expression data, using hierarchical clustering in the *heatmap.2* function from the *gplots* R package (http://CRAN.R-project.org/package=gplots);
- Gene list tables: containing expression values, probe IDs and gene names of the identified outliers.

For the experimental group (Figure 2C):

- Combined heat-maps: containing all identified outliers;
- Combined gene list summary tables: containing all identified outliers showing which individual samples the specific outlier was detected in and thus allowing identification of individuals with common outlier probes.

### Patient Recruitment

These studies were approved by the Joint UCL/UCLH Committee for the Ethics of Human Research (project numbers 02/0324). Forty volunteers were recruited from University College London Hospital (UCLH)/University College London (UCL). Written consent was obtained from all volunteers.

### Monocyte derived macrophage isolation and culture

Peripheral venous blood was collected from subjects into syringes containing 5 U/ml heparin. Mononuclear cells were isolated by differential centrifugation (800 g, 30 min, 20°C) over Lymphoprep and washed twice with sterile phosphate-buffered saline (PBS; GIBCO, Paisley, UK) at 500 g (5 min, 20°C). Cells were resuspended in 10 ml RPMI-1640 medium (Invitrogen) supplemented with 100 U/ml of penicillin (GIBCO), 100 µg/ml streptomycin (GIBCO) and 20 mM HEPES pH 7.4 (Sigma-Aldrich), and plated at a density of approximately 5×10^6^ cells/ml in 8 cm^2^ Nunclon™ Surface tissue culture dishes (Nunc, Roskilde, Denmark) at 37°C, 5% CO_2_. After 2 h, non-adherent cells were discarded and 10 ml of fresh RPMI supplemented with 10% foetal bovine serum (FBS; Sigma) added to each tissue culture dish. Cells were then cultured for 5 days at 37°C, 5% CO2, with the addition of a further 10 ml of fresh 10% FBS/RPMI after 24 h. Cells were then washed twice in PBS, scraped and spun down at 500 g (5 min, 20°C). Cells were resuspended into X-vivo-15 medium (Cambrex, MD, USA) and plated at a density of 10^6^ cells per 8 cm^2^ Nunclon™ dish for a further 25 h at 37°C, 5% CO2.

### RNA purification

Total RNA was prepared from monocyte-derived macrophages, using the RNeasy Mini Kit with RNase–free DNase treatment (Qiagen GmbH, Hilden, Germany). Optical density readings were determined for OD_260_/OD_280_ and OD_260_/OD_230_ using a NanoDrop ND-1000 spectrophotometer (Fisher Scientific, Loughborough, UK) to assess protein and solvent contamination respectively.

### Whole genome microarray analysis

For each sample, 500 ng of total RNA was amplified and purified using the Illumina TotalPrep-96 RNA Amplification kit (Ambion, UK), according to the manufacturer's instructions.

Biotin-Labelled cRNA was then normalised to a concentration of 150 ng/µl and 750 ng was hybridised to Illumina Human-WG6 v3.0 Expression BeadChips (Illumina CA, USA) for 16 h at 58°C. Following hybridisation, beadarrays were washed and stained with streptavidin-Cy3 (GE Healthcare, UK). Beadarrays were scanned using the Beadarray reader and image data was then processed using Genome Studio software (Illumina, CA, USA). The cubic spline normalised data and subject information can be found at http://www.ucl.ac.uk/medicine/molecular-medicine/publications/microarray-outlier-analysis. The microarray data has also been deposited in the Gene Expression Omnibus (GEO) under accession GSE51256.

Due to the relatively low sample sizes, the dataset was tested for normality using the *lillie.test* function from the R-package *nortest* (http://cran.r-projects.org/web/packages/nortest/) [10]. All 20,019 probes on the array were assessed for normality using a p-value of 0.05, followed by correction for multiple testing. This resulted in a very small number of probes (240) found to be not normally distributed indicating that the vast majority of probes are normally distributed and can therefore be used as input to the ZODET method.

### Genomic DNA extraction, PCR verification and sequencing

Peripheral blood samples were collected in an ethylenediaminetetraacetic acid disodium salt vacutainer (BD Bioscience, UK) and gDNA extracted using the QIAamp DNA blood Mini Kit (Qiagen GmbH), in accordance with the manufacturer's instructions. PCR verification of the chromosome translocation involving G-protein receptor-128 (*GPR128*) and TRK-fused gene (*TFG*) was determined using TFG forward primer CCACAGCCTACCTGTGAGTG, *GPR128* reverse primer TGGGTTGTTTGTGGAAAT. Verification of the intact *GPR128* gene was performed using the reverse primer listed for the translocation in combination with forward primer GCAGGCTTTCTTTCTTGAGG. A/G single-nucleotide polymorphism at the exon 7 splice-acceptor site (rs10774671) within 2′-5′-oligoadenylate synthetase 1 (*OAS1*) was determined following the method describes previously [11].

### Generation of Simulated Expression Dataset

A series of simulated expression datasets were generated to contain a total of 20,000 “genes” (or probes) and 100 samples. The samples were divided into equally sized groups of 50 control samples and 50 experimental samples, with the gene expression values for each group generated from a standard normal distribution (i.e., with a mean value of 0 and standard deviation of 1). In order to simulate example cases of gene outliers a range of genes were randomly selected to contain an equal number (20, 50, 100, 250, 500, and 1000) of up- and down-regulated patient samples relative to the control group. For each of these simulated outlier genes, we randomly assigned a number of samples from the experimental test group (between 5 and 10) to correspond to expression levels significantly different to the standard normal distributions. These expression levels were randomly assigned so as to correspond to p-values of being part of the standardised normal sample distributions of between 0.05 and 1×10^−6^.

## Results

### Comparison to Existing Gene Outlier Detection Methods

Recently, the GTI outlier detection algorithm was described and compared to other existing gene expression outlier detection methods [4]. It was shown that the GTI method performs comparably to other outlier detection methods (including: cancer outlier profile analysis (COPA); outlier sums (OS); and the outlier robust t-statistic (ORT)) when using a simulated expression dataset [5], [6], [12]. In order to benchmark our z-score based outlier detection method ZODET against these existing methods we performed a similar comparison to the GTI method, using both simulated expression datasets and also an expression dataset compiled from a cohort of human monocyte-derived macrophage samples split into two equal sized groups.

Both the ZODET and the GTI methods were run on the simulated expression datasets of 100 samples and 20,000 probes and the resulting outputs compared. ZODET was run with the default p-value detection method and threshold, of 0.01, and a fold-change threshold of 0.5. The GTI method was also run with default settings (except that an automatic log transformation of the expression values was not carried out).

**Figure 3:**
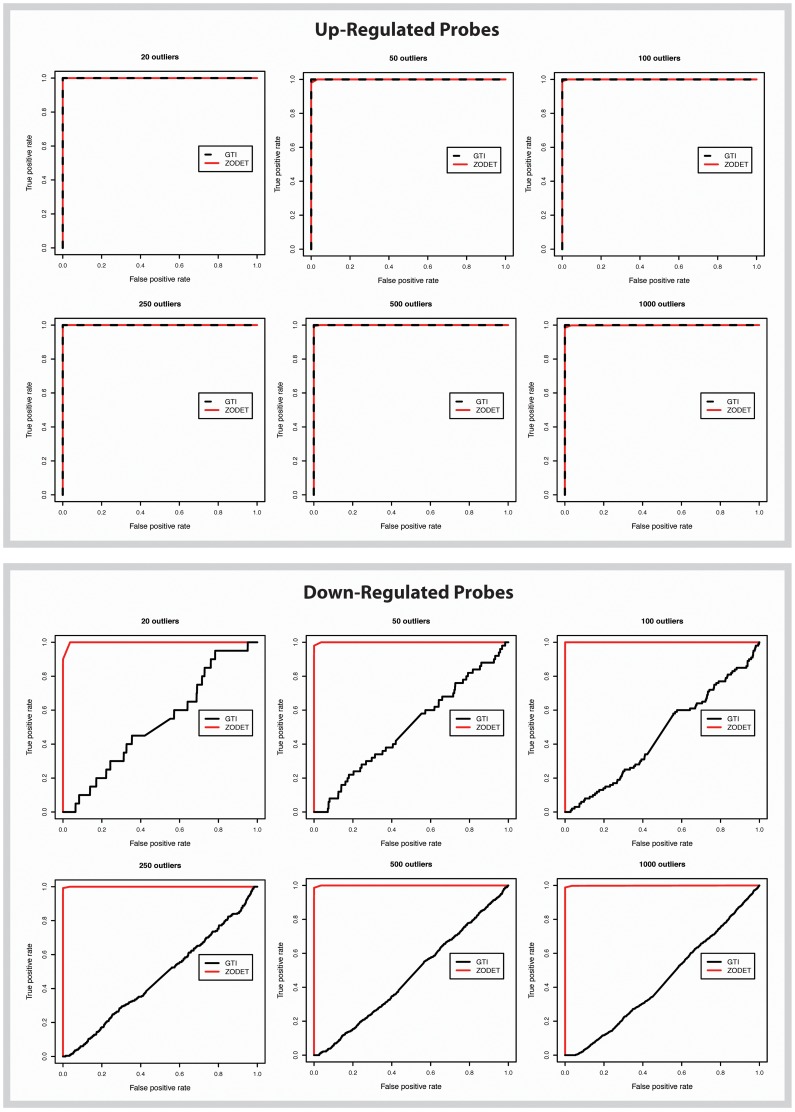
Receiver operating characteristic (ROC) curves for ZODET and GTI outlier detection. ROC curves are plotted based on simulated outliers (for a range of up- and down-regulated probes) from a dataset of 20,000 probes.

To compare the ability of the two methods to correctly identify probes that correspond to the simulated outlier expression values we used Receiver Operating Characteristic (ROC) curves on the ranked outputs (Figure 3). The GTI output was ranked in descending “GTI score” order and ZODET was ranked using the number of samples that were detected to be associated with an outlier probe. These methods of ranking highlight an important difference between outlier detection using ZODET and the GTI method; that is, the GTI method only detects whether a particular gene (or microarray probe) contains outlier samples, whereas ZODET detects the likelihood that a gene (or probe) is an outlier for each of the individual test samples. Therefore, ZODET has the advantage of being able to identify both the genes and the specific test experimental samples that are significantly associated with outlier gene expression values.

Interestingly, both methods consistently performed well when identifying the up-regulated true positive outliers in the simulated expression dataset, resulting in an area under the ROC curve (AUC) of 1.0 (Figure 3). When identifying the down-regulated outliers, ZODET, clearly out-performed the GTI method, with average AUC values of 0.998 and 0.476, respectively (Figure 3). This was expected because the GTI method is specifically designed to identify outliers that are over-expressed relative to the control group, whereas ZODET detects both under- and over-expressed outliers equally well.

In summary, the two methods performed equivalently when identifying the up-regulated outlier genes from the simulated dataset, but importantly ZODET is also able to detect down-regulated outliers to a high level of accuracy.

### Identification of outlier genes in a monocyte-derived macrophage microarray dataset

An example microarray dataset is available on our webpage (http://www.ucl.ac.uk/medicine/molecular-medicine/publications/microarray-outlier-analysis). This dataset consists of two data files: (i) an “*EXPRESSION DATA*” file - containing the microarray expression data from monocyte-derived macrophages collected from 40 volunteers split into two equal sized groups labeled control (A1 to 20) and experimental (B1 to 20); and (ii) an “*EXPERIMENT INFORMATION*” file - containing associated experimental parameters. The technical specifications of these input files are described in the provided software documentation.

A ZODET analysis was performed with the thresholds set at a log2 fold-change >1.75 and unadjusted p-value <0.0001. Combining the ZODET analysis results from subjects B1 to B20 identified 19 up- and 14 down-regulated probes in this population (Table 1). Three probes corresponding to two genes were identified as significantly up-regulated in three or more individuals within the experimental cohort. Three individuals were found to over-express either G-protein coupled receptor 128 (*GPR128*) (ILMN_2125395 and ILMN_1808078) or chemokine (C-C motif) ligand 3-like 1 (*CCL3L1*)(ILMN_1773245). Two probes corresponding to two genes were found to be down-regulated in three or more individuals in the experimental population. 2′-5′-oligoadenylate synthetase 1 (*OAS1*) (ILMN_1658247) and ribosomal protein S23 (*RPS23*) (ILMN_1772459) were significantly attenuated in six and three of the twenty subjects tested, respectively. However, the probe for *RPS23* (ILNM_1772459) contains a recognised single nucleotide polymorphism (SNP) (rs3738) which accounts for the three down-regulated outliers identified in the experimental cohort. The SNP results in poor probe hybridisation and not a genuine reduction in gene expression. PCR verification should be conducted on all identified probes in order to discount false positives resulting from poor probe hybridisation.

**Table 1:** Combined ZODET analysis and corresponding GTI results from 20 experimental subjects.

Probe ID	Symbol	Count	GTI Score	GTI Rank
**Up-Regulated Probes (p<0.0001, fc>1.75)**				
ILMN_2125395	GPR128	3	4.889	1
ILMN_1808078	GPR128	3	4.455	2
ILMN_1773245	CCL3L1	3	2.46	12
ILMN_1732198	UTS2	2	3.862	3
ILMN_1661861	CSF2	1	−0.062	15671
ILMN_1668134	GSTM1	1	1.653	52
ILMN_1671818	UTS2	1	3.223	6
ILMN_1676256	TPSAB1	1	1.453	70
ILMN_1682775	EDN1	1	1.293	104
ILMN_1688423	FCER1A	1	0.446	1585
ILMN_1693269	GNG8	1	0.644	746
ILMN_1710186	CCL17	1	1.17	143
ILMN_1740418	CYP27B1	1	1.087	179
ILMN_1752965	GREM1	1	2.621	11
ILMN_1784532	COL22A1	1	2.995	8
ILMN_2100209	CCL4L1	1	1.661	51
ILMN_2169801	TPSAB1	1	2.454	13
ILMN_2289593	FXYD2	1	1.279	111
ILMN_2294762	AMY1A	1	0.727	579
**Down-Regulated Probes (p<0.0001, fc>1.75)**				
ILMN_1658247	OAS1	6	−0.758	22220
ILMN_1772459	RPS23	3	−0.194	20017
ILMN_1682928	CPVL	1	0.288	3177
ILMN_1694400	MSR1	1	0.601	858
ILMN_1704291	LOC645317	1	0.068	9180
ILMN_1721035	MS4A6A	1	0.501	1281
ILMN_1722622	CD163	1	0.931	302
ILMN_1764709	MAFB	1	0.553	1027
ILMN_1777190	CFD	1	0.621	810
ILMN_1797731	MS4A6A	1	0.197	4894
ILMN_2054607	CYP4V2	1	0.216	4466
ILMN_2101278	RGS18	1	0.12	7121
ILMN_2359800	MS4A6A	1	1.153	152
ILMN_2379599	CD163	1	0.753	525

The GTI ranking is based on the 22,375 probes analysed. ZODET thresholds were set at p<0.0001 and fold change >1.75. All identified probes are shown along with, the gene symbol, the number of individuals who were classified as outliers, the GTI score and GTI rank. The “count” shows the number of individuals from the experimental group that were classified as outliers with respect to the control group for each probe. The 19 up-regulated probes and 14 down-regulated probes are shown.

A GTI analysis was also performed on the same data set and the results compared to the output from ZODET (Table 1). In accordance with the simulated data analysis, the GTI software was only capable of identifying the up-regulated probes and gave the highest ranking to both of the GPR128 probes which were also identified using ZODET.

**Figure 4:**
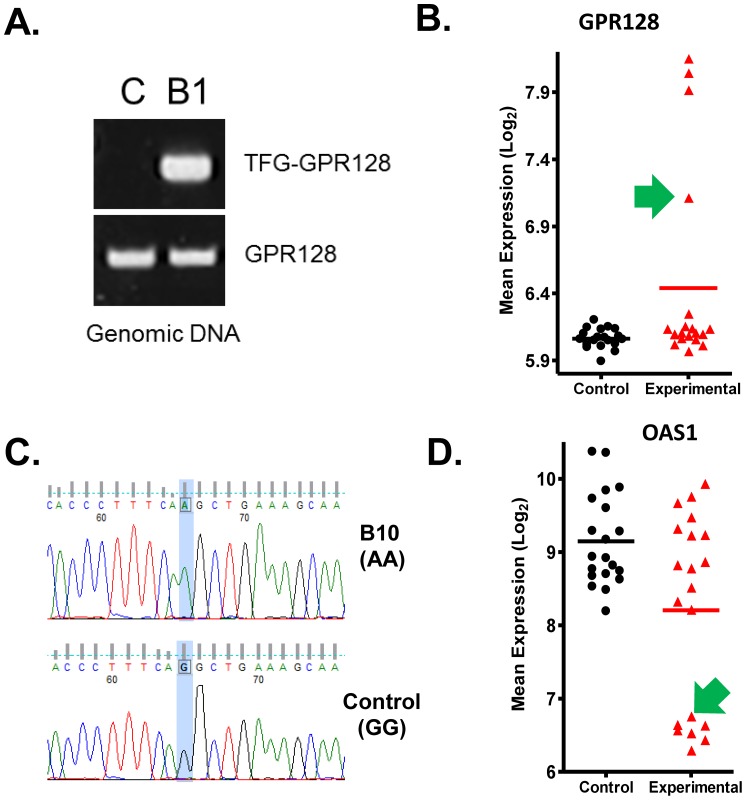
Experimental validation of two genes identified by ZODET. **A.** PCR verification of the *TFG-GPR128* translocation in individual B1 who was identified as an outlier for *GPR128* using ZODET analysis. **B.** Expression profile for the *GPR128* probe (ILMN_2125395) in controls and experimental subjects. Green arrow identifies an individual who carries the *TFG-GPR128* translocation, demonstrates elevated *GFP128* levels but was not identified by the ZODET analysis using the p<0.0001 and fold change >1.75 thresholds. **C.** Genomic DNA sequencing of the *OAS1* gene revealed a splice site polymorphism A>G (rs10774671) in all individuals who were identified as outliers by the ZODET analysis. All six individuals were shown to be homozygous for the polymorphism. **D.** Expression profile for the *OAS1* probe (ILMN_1658247) in controls and experimental subjects. Green arrow identifies an individual who carries the splice site polymorphism, demonstrates reduced *OAS1* levels but was not identified by the ZODET analysis using the p<0.0001 and fold change >1.75 thresholds.

In order to determine if the abnormal gene expression results from genetic mutations, both *GPR128* and *OAS1* genes were sequenced in the subjects identified as outliers. No mutations were found in any of the individuals who over-expressed *GPR128* (data not shown). However, a previous report identified a chromosome translocation involving *GPR128* and TRK-fused gene (*TFG*) which results in the generation of an in-frame *TFG-GPR128* fusion transcript [13]. This transcript was found to be constitutively expressed in all tissues and could be the possible cause of *GPR128* over expression identified in these individuals. PCR analysis and sequencing was performed on the three outlier subjects (Figure 4A). The results confirmed the presence of the *TFG-GPR128* translocation in the three patients identified as outliers for the *GPR128* gene. Further PCR analysis was carried out on the remaining 17 experimental subjects and 20 controls. One additional experimental subject was found to carry the translocation and from the expression data the increased levels of GPR128 can be seen but the thresholds applied to the ZODET analysis meant that this individual was not identified as an outlier (Figure 4B, green arrow). None of the control subjects carried the translocation. Therefore the ZODET analysis identified three out of the four experimental subjects that were carriers of the TFG-GPR128 translocation. The most common under-expressed outlier gene was *OAS1* which was found in six experimental individuals. The gene was sequenced in all six subjects and was found to be homozygous for a known A/G single-nucleotide polymorphism at the exon 7 splice-acceptor site (rs10774671) in the *OAS1* gene (Figure 4C) [11]. Further sequencing of the remaining experimental subjects identified an additional individual who was homozygous for the rs10774671 polymorphism (Figure 4D, green arrow). Therefore, the ZODET analysis identified six of the seven individuals within the experimental cohort that expressed an *OAS1* splice variant. In summary, the ZODET analysis identified a number of outlier genes within the experimental population two of which we were able to show resulted from a genetic abnormality. GTI identified the translocation event but missed the splice site polymorphism which resulted in attenuated gene expression and was present in 40% of the experimental population. As these abnormalities were only present in a subset of the patients in the experimental group, standard group-based statistics (e.g. a t-test) would fail to identify a significant difference. These findings demonstrate a potential advantage of the ZODET analysis over the currently available software.

## Discussion

We have described software designed for the identification, analysis and visualisation of outlier genes and individual outlier samples in microarray expression data via a user-friendly GUI. This method implements a z-score based measure of the level of deviation of individual microarray samples, for a particular gene, from an assumed normal distribution of control comparison samples. This differs in approach from the GTI method, which calculates a score for each gene related to the proportion of samples in a specific group that have gene expression levels above a defined statistical outlier threshold. An important advantage of the ZODET software is the user-friendly implementation of a z-score based outlier detection method, which has been specifically designed to be used by individuals with limited bioinformatic experience through a GUI.

Since the GTI method was previously shown to perform comparably to other outlier detection methods we benchmarked the performance of ZODET to this method [4]. We have shown that ZODET performs comparably to the GTI method when identifying up-regulated outliers in a large simulated expression dataset containing a defined numbers of outliers at varying levels of statistical significance from the standardised population mean. ZODET was also shown to perform with a very high accuracy and very low false positive rate when identifying genes and samples simulated to represent down-regulated outliers.

In addition to outlier analysis, other methods that use gene expression variability between groups can be used to identify genes that may have biological relevance [14]–[17]. However, in general for these approaches a more robust and dynamic change in expression is required, whereas outlier analysis methods, such as ZODET, can identify an individual or small sub-groups within the experimental population.

We have also demonstrated the power of the method for detecting outlier genes of biological interest in a dataset containing gene expression profiles from a cohort of primary monocyte derived macrophages isolated from human subjects. In particular, the most common under- and over-expressed genes identified by ZODET were shown to result from germline mutations. A functional consequence resulting from the *TFG*-*GPR128* translocation has so far not been reported. Whereas, a previous report has shown that the *OAS1* splice site mutation results in the loss of the commonly expressed OAS1 enzyme (p46) and the generation of a dual-function antiviral/proapoptotic isoform (p48) and a novel isoform (p52) with no known role. (11) The fact that these genetic alterations can be identified provides evidence that this form of analysis could be of benefit in the study of complex heterogeneous diseases. Proof that this form of analysis can provide disease- relevant findings comes from the identification of the fusion of *TMPRSS2* and *ETS* transcription factor genes in prostate cancer through the use of the COPA software [3].

Of particular note was the ability of ZODET to identify outlier genes that were either significantly up- or down-regulated when compared to the controls. ZODET is also capable of identifying an individual gene expression abnormality in one experimental subject, which may allow the identification of rare genetic events that have extremely high disease penetrance. These properties provide an important addition to other commonly used outlier detection methods, such as COPA and GTI, which concentrate solely on the identification of strongly up-regulated or amplified genes in subgroups of patients. Each of these outlier detection methods has advantages and disadvantages and they could well be most effectively utilised in combination with each other, although we have not attempted to do so in this study.
